# Impact of sky view factor on multimodal physiological responses: integrating EEG, heart rate, and thermal sensation in urban landscapes

**DOI:** 10.1038/s41598-026-58340-1

**Published:** 2026-06-16

**Authors:** Lei Fan, Zixian Li, Lu Chang, Yan Zhou

**Affiliations:** 1 Forestry College, Shenyang Agricultural University, Shenyang, 110866 China; 2 Key Laboratory of Northern Landscape Plants and Regional Landscape, Shenyang, 110866 China

**Keywords:** Sky view factor, Outdoor, Thermal comfort, EEG, Heart rate variability, Neuro-environmental perception, Neuroscience, Physiology

## Abstract

Traditional thermal comfort models often fail to capture the dynamic neurophysiological complexity of outdoor human perception. This study introduces a neurophysiological approach to investigate how Sky View Factor (SVF) modulates human comfort. A controlled indoor-outdoor protocol was implemented, transitioning participants from a neutral baseline to a stressor before exposure to varying SVF gradients. High-precision EEG, HRV, and skin temperature data were synchronously analyzed. Our primary analysis did not reveal a statistically significant main effect of SVF on alpha power (F(2, 38) = 1.89, *p* = 0.165, η^2^ = 0.090). Heart rate also did not significantly change from baseline to stress (t(40) = 0.40, *p* = 0.694). However, a marginal trend was observed for higher alpha power in High SVF compared to Medium SVF environments (Welch’s t(21) = 1.96, *p* = 0.065), suggesting a potential pattern that warrants further investigation with larger samples. These findings suggest that while the visual openness associated with high SVF may function as a psychological buffer against thermal stress, this effect is embedded within a composite environmental context where SVF co-varies with microclimatic factors like solar radiation.

## Introduction

Thermal comfort research has long been dominated by physical and physiological models, most notably Fanger’s Predicted Mean Vote (PMV) and Predicted Percentage Dissatisfied (PPD) indices, which are based on heat-balance equations under steady-state laboratory conditions. While the Fanger’s PMV-PPD model provides a foundational framework for indoor steady-state environments, its applicability to transient and complex outdoor settings is limited due to the physiological and psychological adaptation of individuals. Current research in outdoor thermal comfort (OTC) has shifted towards adaptive models that account for microclimatic variability and individual differences. While these models have provided a fundamental framework for assessing indoor environments, their applicability diminishes in dynamic, multi-stimulus outdoor settings such as urban parks and landscaped gardens^1,2^. A primary limitation lies in their treatment of the human body as a passive thermodynamic entity, largely ignoring the central role of the central nervous system (CNS) in integrating environmental stimuli, physiological signals, and psychological states to form ultimate thermal perception.

The emerging field of Environmental Neuroscience addresses this gap by demonstrating how environmentally shaped cognitive and emotional processes directly influence perception and behavior^3^. Indeed, thermal sensation is not merely physiological but a complex neurophysiological process, engaging cortical and subcortical regions like the hypothalamus, insula, and prefrontal cortex to evaluate thermal and non-thermal inputs. Research confirms that visual, auditory, and olfactory stimuli can modulate thermal comfort independent of physical temperature^4,5^. Specifically, viewing natural landscapes correlates with reduced stress and heightened parasympathetic activity, suggesting a pathway for green environments to alleviate thermal discomfort. Recent studies further indicate that the naturalness of the visual environment itself is a potent modulator; for instance, Zhao et al.^6^ found that street visual environments assessed through physiological feedback and deep learning impact young adults’ emotional well-being, providing novel insights into the neurological benefits of green landscapes.​

Sky View Factor (SVF)—the ratio of visible sky to the total hemisphere at a given point—is a key parameter capturing urban geometry and vegetation density. Although its microclimatic impacts (e.g., on solar radiation) are well-known^7,8^, its role as a visual modulator of neural and physiological states remains unclear. While SVF is known to affect pedestrian thermal comfort^2,8^, existing research focuses on environmental physics and standard physiology, neglecting the underlying neurophysiological mechanisms. Notably, Wang et al.^8^ exemplified the multifactorial fusion trend, revealing that SVF simultaneously influences pedestrians’ thermal sensation and air quality perception, with significant interaction effects (e.g., increased pollution reduces tolerance to high temperatures), highlighting the need for systematic thinking in urban design to balance shading, ventilation, and pollutant dispersion in green spaces.​While SVF is known to affect pedestrian thermal comfort primarily through its modulation of solar radiation and mean radiant temperature (Tmrt), its role as a composite visual-thermal modulator of neural and physiological states remains unclear. It is crucial to recognize that in real-world settings, changes in SVF inherently alter both visual exposure and microclimatic parameters. Therefore, any observed “neural pathway” should be interpreted as an integrated brain response to this combined visual-thermal cue, rather than a purely isolated visual effect.

Electroencephalography (EEG) advances now enable decoding of brain dynamics in environmental contexts. Studies have linked EEG biomarkers (e.g., theta/gamma oscillations) to thermal discomfort and emotion^5,9^, while HRV and skin temperature reliably indicate autonomic nervous system activity during relevant stimuli^10,11^. A systematic review of 44 studies (2000–2023) conducted by our team (data not shown) indicated that EEG applications in this field have grown significantly (68.18% post-2020), with a stable correlation between thermal comfort and EEG frequency domain features: improved comfort correlates with enhanced low-frequency oscillations (θ, α) and weakened high-frequency oscillations (β), with the temporal and occipital lobes being key brain regions^9^. While many EEG studies have traditionally been conducted in laboratory-controlled environments to minimize noise and standardize experimental conditions, there is a notable and increasing trend toward mobile and real-world EEG applications that enhance ecological validity^12,13^. This shift is driven by the recognition that laboratory settings, despite offering high control, often limit the generalizability of findings to real-life situations^12,14^. Nevertheless, an integrated approach combining high-density EEG with physiological and subjective metrics to decipher “brain-body-environment" interactions in real-world landscapes is still lacking. Most EEG research (86.36%) remains confined to laboratories, with only one study focused on practical application, highlighting a significant gap in validation within real architectural or urban environments^9^. Despite the extensive exploration of SVF in urban heat island studies, a critical research gap remains: the underlying neurophysiological mechanism by which SVF-induced visual stimuli interact with thermal perception is not yet fully understood. Most existing studies rely on subjective scales, which are prone to recall bias. There is a pressing need for multimodal evidence—integrating central (EEG) and peripheral (HRV, skin temperature) signals—to quantify the human–environment interaction in real-world landscape settings.

To bridge this existing gap, a multimodal neurophysiological framework is proposed to examine how SVF-modulated visual features in gardens influence thermal perception through both central and peripheral pathways. A controlled experimental protocol was designed to simulate a realistic environmental sequence, transitioning from a neutral indoor baseline to an emotional visual stressor, and finally to outdoor green spaces characterized by varying SVF gradients. During this process, environmental parameters, 14-channel EEG signals, multi-site skin temperatures, HRV indices, and subjective evaluations were synchronously collected.

The core objective of this research is to elucidate the cross-system coupling mechanism of the “EEG-Skin-Heart Rate” axis under SVF-modulated visual exposure. Specifically, the following dimensions are examined:

1. Whether and how SVF regulates neural oscillatory patterns (e.g., alpha, beta, and gamma bands);
2. How visual and thermal pathways—referring here to the processing of visual scene openness via the retino-cortical pathway and the processing of thermosensory information via spinothalamic and thermosensory cortical pathways—interact at the level of the autonomic and central nervous systems;
3. Clarify the coupling patterns of neural responses (EEG) and physiological indicators (heart rate/skin temperature) in the SVF-thermal perception relationship, and investigate the co-occurrence patterns.

By revealing the neural correlates of environmental perception, this work contributes to a paradigm shift from a deterministic “environment-physiology” model toward an integrative “environment-neuro-physio-psychology” framework. Our findings provide novel insights for developing brain-informed urban designs and precision landscape interventions that enhance human well-being in a warming climate.

## Methods

### Study area

According to the Code for Thermal Design of Civil Buildings, Shenyang is classified as belonging to a severe cold region (2B zone). Located between 41° 11′–43° 02’ N and 122° 25′–123° 48′ E, the city experiences a typical cold temperate continental monsoon climate (Köppen Dwa), characterized by four distinct seasons, short transitional periods in spring and autumn, and long, cold, dry winters (Table 1).

**Table 1:** Climatic characteristics of Shenyang (Severe Cold Region, 2B Zone).

Parameter	Value
Climate Classification (Köppen)	Dwa (Cold, Continental Monsoon)
Average Ta (Coldest month, Jan)	− 12.0 °C
Average Ta (Hottest month, Jul)	25.5 °C
Annual Temperature Range	42.3 °C
Extreme Ta (Winter/Summer)	− 29 °C/38 °C
Highest Monthly Avg RH	76% (Jul), 78% (Aug)
Peak Solar Radiation Period	April–June
Clear-sky days in Winter	72%
Annual Mean Wind Speed	2.65–2.67 m/s
Calm conditions (wind < 1.5 m/s)	89% frequency

Meteorological data were obtained from the Meteorological Dataset for Thermal Environment Analysis of Buildings in China. The seasonal mean relative humidity is 35.2% in winter and 38.6% in summer. Solar radiation peaks between April and June, ranking among the highest annual levels, but declines in July. During winter, clear-sky days account for 72% of the season, with direct solar radiation intensity being 15–20% higher than in Beijing. Wind speed also exhibits strong seasonal variation, with spring (March–May) being the windiest season (reaching 3.60 m/s in April) and summer the calmest (as low as 2.00 m/s in August). The annual mean wind speed ranges from 2.65 to 2.67 m/s, while calm conditions (wind speed < 1.5 m/s) occur with a frequency as high as 89%.

### Field study design

To systematically investigate the impact of sky exposure, five representative landscape spaces were selected as outdoor experimental sites (Table 2). SVF values were captured at a height of 1.5 m using a Canon EOS 6D DSLR with a Sigma 8 mm fisheye lens and calculated via RayMan software.

**Table 2:** Description of 5 selected spaces.

Site code	Site type	Summer SVF	Thermal environment characteristics	Summer fish-eye photo
PL	Sports plaza (fully open)	1.000	A fully open sports field with sparse trees around the track, offering unobstructed views of the sky.	
AT	Front square (deciduous trees)	0.846	A plaza in front of the building, surrounded by a few trees, with moderate greening and mainly hard paving, making it a semi-open space.	
SQ	Garden plaza (mixed vegetation)	0.716	Dense trees surround the area, but the sky is quite open, with a landscape water pool nearby, also considered a semi-open space.	
PA	Landscape pavilion (structure)	0.142	The space under the pavilion is open without shade, but it is surrounded by greenery, providing a fairly open view, mainly with hard paving.	
TS	Shaded square (mixed vegetation)	0.073	The area is densely greened, surrounded by tall trees and shrubs, featuring a pavilion and wooden paving, providing excellent shade.	

For robust statistical interpretation, the SVF gradient was categorized into three distinct levels using an equidistant interval approach across the sampled range. The classification criteria were defined as: Low SVF (0.0721 < SVF ≤ 0.258), representing densely shaded forest environments (Sites TS and PA); Medium SVF (0.629 < SVF ≤ 0.815), representing semi-open transitional landscapes (Site SQ); and High SVF (0.815 < SVF ≤ 1)), representing fully open urban squares (Sites AT and PL). This 'polar-contrast’ sampling, supplemented by the transitional medium-gradient site, provides an explicit framework for capturing threshold effects in neurophysiological and thermal responses. By encompassing these typical extremes, the selected scenarios effectively represent the diverse urban sky exposure experienced by residents in cold-region cities.

Indoor experiments (Stage 1 & 2) were conducted in a laboratory equipped with artificial climate control. The initial environmental settings of the lab were set to a thermal neutral temperature (24–26 °C), relative humidity controlled at 50% ± 10%, and wind speed less than 0.1 m/s, to ensure that the subjects were in thermal equilibrium at the start of the experiment.

To investigate the OTC conditions in Shenyang’s Dwa climate zone, a mobile synchronous monitoring method was adopted. Five outdoor spaces with distinct SVF gradients were selected to represent typical urban forms, including a plaza, pocket park, corridor pavilion, shaded grove, and open square. Trained researchers carried portable devices and conducted circular measurements across preset stations. Each measurement point was monitored for 30 min, with a full cycle lasting approximately 2.5 h.

All environmental monitoring instruments were fixed at a height of 1.5 m above ground level to simulate the average breathing zone and visual field height of a standing adult. Microclimatic parameters were recorded in real-time using a suite of high-precision devices. Specifically, air temperature (Ta), relative humidity (RH), and wind speed (V) were measured using a Kestrel 4500 handheld weather station, which provides an accuracy of ± 0.5 °C for temperature, ± 3% for humidity, and ± 0.1 m/s for wind speed. Solar radiation (G) was captured by a TES 1333 pyranometer with a measurement accuracy of ± 5%. To facilitate the calculation of the Mean Radiant Temperature (MRT), globe temperature (Tg) was monitored using a WBGT-213B globe thermometer with an accuracy of ± 2 °C. All environmental data were synchronized at 1-min intervals to ensure temporal consistency with physiological recordings.

The questionnaire consisted of three sections (Appendix 1). The first section focused on personal information, including gender, age, height, and weight^15,16^. Duration of residence in the city was also included and categorized into three groups for data analysis: less than 1 year, 1–4 years, and more than 4 years.

**Fig. 1:**
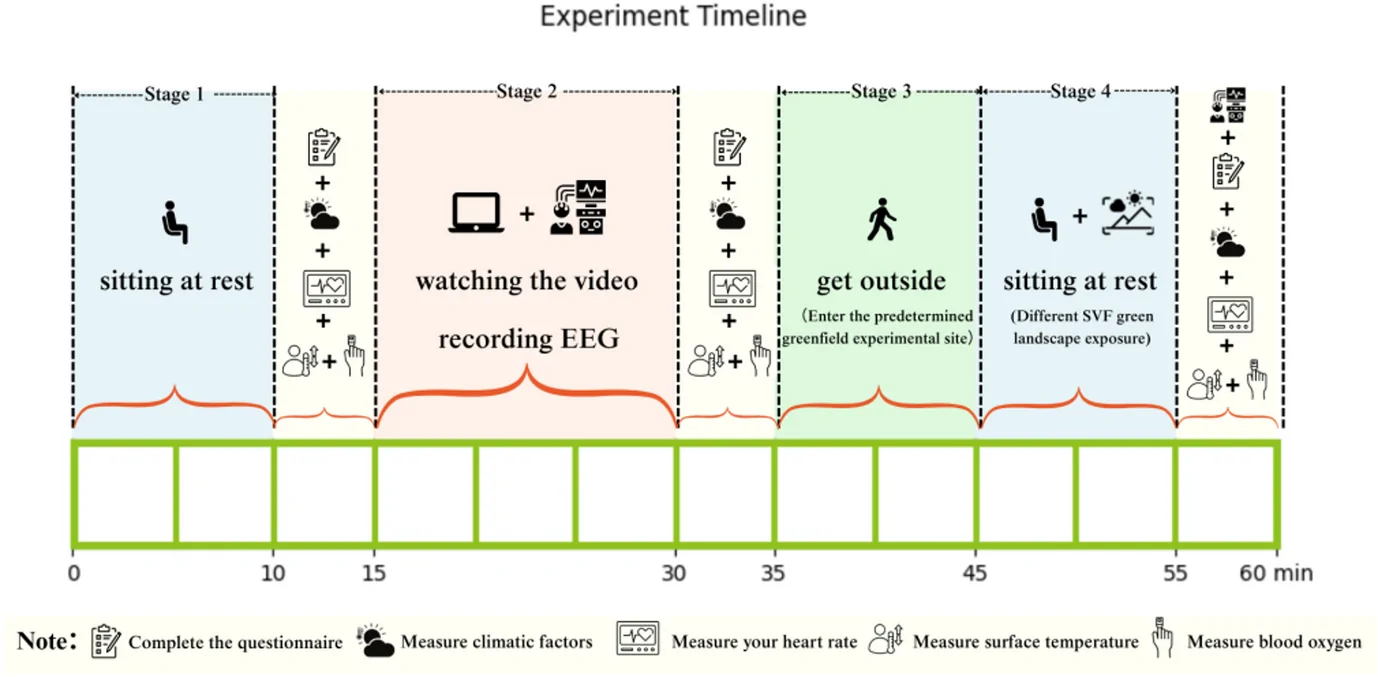
Experiment timeline for thermal comfort and physiological measurements.

The second section investigated thermal perception through three indices: thermal sensation vote (TSV), thermal comfort vote (TCV), and thermal preference vote (TPV). TSV referred to the subjective thermal sensation as reported by participants^17^, and followed the 7-point ASHRAE scale ranging from cold (− 3) to hot (+ 3) (ANSI, 2017). TCV assessed the perceived comfort level using a 3-point scale (uncomfortable, acceptable, comfortable), while TPV captured respondents’ desired thermal state based on the 3-point McIntyre scale. The third part assesses emotional state using the Chinese version of the Positive and Negative Affect Schedule (PANAS), evaluating participants’ emotional experiences at a specific moment. Figure 1 shows the details of all subjective assessment indicators of the thermal environment (TSV, TCV, TPV, and PANAS).

A priori power analysis using G*Power 3.1 for a repeated-measures ANOVA (within factors) indicated that a total sample size of 36 was required to achieve a power of 0.80, assuming a medium effect size (f = 0.25), an alpha of 0.05, and a correlation of 0.5 among repeated measures. Our recruitment of 40 healthy university students (20 males and 20 females; mean age 22.3 ± 1.5 years; mean BMI 21.6 ± 2.1 kg/m^2^) exceeded this requirement, ensuring adequate statistical power. To minimize inter-individual thermal variance, all participants were required to wear standardized clothing consisting of a short-sleeved T-shirt and long trousers, with an estimated total clothing insulation (Icl) ranging from 0.5 to 0.7 clo.

Potential participants were excluded if they reported histories of cardiovascular or neurological disorders, abnormal thermoregulation, or uncorrected visual impairment. Those included were instructed to maintain regular sleep patterns and abstain from caffeine, alcohol, or substances affecting the central nervous system for at least 24 h prior to testing. All protocols were conducted in accordance with relevant guidelines and approved by the Ethics Committee of Human Studies, College of Forestry, Shenyang Agricultural University (Approval No. CF-EC-2025-234), with written informed consent obtained from all subjects prior to participation.

The study employed a controlled indoor-outdoor sequential protocol designed to simulate real-life transitions among environments with differing thermal and visual characteristics (Fig. 1).To ensure data integrity and prevent carry-over effects from one environment to the next, each of the five SVF scenarios was treated as a separate experimental trial. Participants completed a full “baseline-stress-exposure-recovery” sequence for one SVF site before starting the sequence for the next site on a different day. A single complete trial commenced with a 10-min Indoor Baseline phase, where participants remained seated in a thermally neutral environment (air temperature: 24–26 °C; relative humidity: 50 ± 10%; wind speed < 0.1 m/s) to stabilize their initial physiological state. This was followed by a 5-min Visual Stress Intervention; while maintaining the same physical setting, participants viewed a standardized 5-min audiovisual stimulus: a red-tinted video depicting a bustling, overcrowded urban intersection with high-density pedestrian flow, chaotic traffic noise, and intermittent sharp sounds (e.g., car horns and emergency sirens). The effectiveness of this stress induction was verified by a significant increase in self-reported negative affect (PANAS) and a significant increase in heart rate compared to the baseline phase.

Subsequently, participants were escorted to one of the five pre-selected outdoor sites for a 15-min Outdoor Green Space Exposure, representing a specific SVF gradient ranging from 0.073 to 1.000. Upon completion of the multimodal data collection at an outdoor site, participants were immediately led back to the controlled indoor environment for a mandatory recovery period. This transition ensured that the physiological and psychological markers returned to baseline levels before the next experimental cycle began. Each participant completed all five SVF scenarios through this repetitive “baseline-stress-exposure-recovery” sequence, with the order of sites randomized to mitigate potential order bias. As illustrated in Fig. 1, EEG and peripheral physiological data (HR, skin temperature) were not recorded continuously throughout the entire session. Instead, EEG acquisition was limited to two discrete time windows: (1) the entire 15-min Visual Stress Intervention phase (Stage 2) , and (2) a 5-min period immediately following the 10-min outdoor recovery phase (Stage 4) . Specifically, after a 10-min baseline rest and a 5-min baseline measurement (no EEG recording), participants underwent a 15-min stress phase with continuous EEG. Following a 5-min post-stress assessment and a 10-min walk to the outdoor site, they experienced a 10-min outdoor recovery (no EEG), after which a final 5-min monitoring session (including EEG) was conducted.

The experimental durations were carefully calibrated based on physiological stabilization requirements: the 10-min indoor baseline ensured participants reached a steady cardiovascular and thermal state; the 5-min stress induction (via standardized video) was designed to establish a consistent psychological starting point; and the 15-min outdoor exposure period was selected to allow sufficient time for the human body to achieve a quasi-steady thermal equilibrium while maintaining the stability of EEG signal acquisition. The total duration per participant was approximately 60 min. All experiments were conducted during the spring and summer of 2025.

**Fig. 2:**
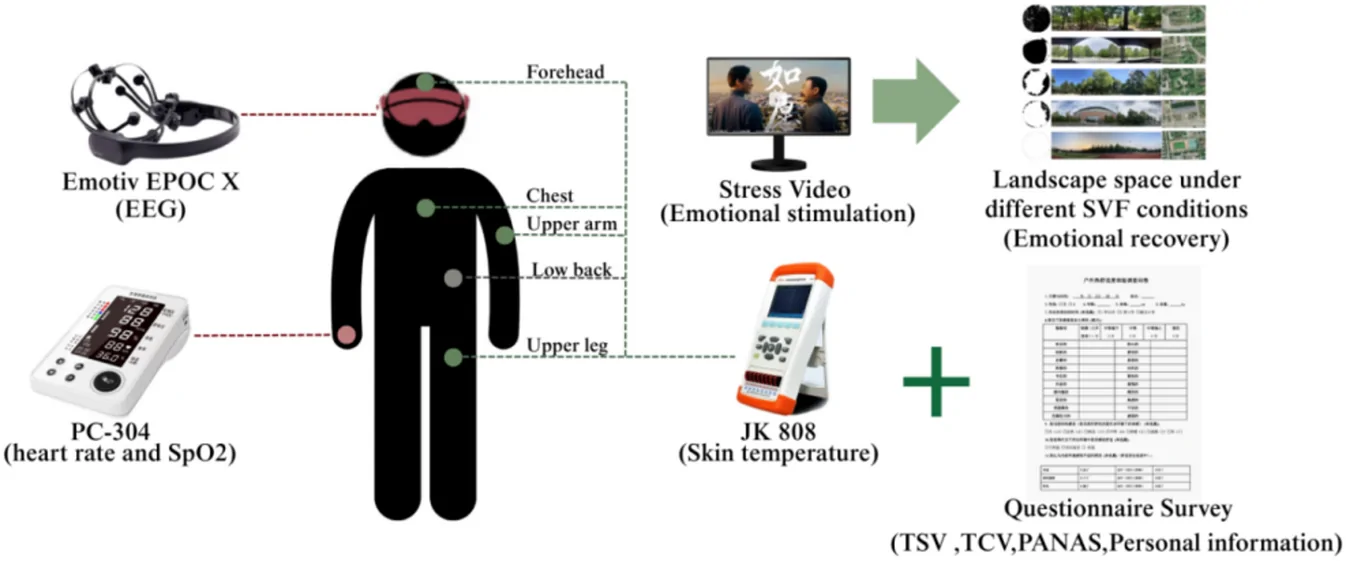
Experimental procedure for monitoring emotional change and data acquisition.

The neurophysiological response monitoring system integrated high-fidelity brain activity tracking with peripheral physiological sensing to capture a holistic “brain-body” profile (Fig. 2). Specifically, cortical dynamics were recorded via a 14-channel wireless EEG system (Emotiv EPOC X), with electrodes configured according to the international 10–20 system. The 14 channels included: AF3, F7, F3, FC5, T7, P7, O1, O2, P8, T8, FC6, F4, F8, AF4. Data were digitized at a sampling rate of 128 Hz. Concurrent with the EEG monitoring, systemic physiological indicators—including heart rate (HR), heart rate variability (HRV), and blood oxygen saturation (SpO₂)—were captured through a precision finger pulse oximeter. To account for localized thermal dynamics, wireless sensors were deployed to record skin temperature across five strategic anatomical sites: the chest, arm, thigh, back, and forehead. This objective dataset was complemented by immediate subjective assessments following each experimental phase. Participants reported their TSV and TCV alongside their emotional state, the latter of which was quantified using the PANAS. By synchronizing these multidimensional metrics, the framework effectively bridged the gap between subconscious neural oscillations and conscious environmental perception.

### Thermal index calculation

Physiological Equivalent Temperature (PET) was calculated using the RayMan Pro 3.1 software, a microscale radiation model developed by the Chair of Meteorology and Climatology at Albert Ludwigs University of Freiburg. The model is based on the original RayMan model developed by Matzarakis et al.^18^. Tmrt and PET outputs were generated from input variables including air temperature, relative humidity, wind speed, solar radiation, SVF, personal clothing and activity parameters. This software has been widely applied in OTC studies^19–21^. The input parameters included meteorological data (Ta, RH, Va, Tmrt), personal data (age, height, weight, clothing, and activity), and geographical data (longitude, latitude, and altitude).

UTCI was computed using the Pythermalcomfort package, an efficient and accurate Python library developed by Tartarini at the University of California, Berkeley^17^. The inputs to this package were Ta, RH, Va, and Tmrt. Notably, Tmrt was calculated according to Eq. (1) in ISO 7726. Tg, Ta, and Va were obtained from field measurements. In this study, the globe emissivity (ε) was assumed to be 0.95, and the globe diameter (D) was set to 150 mm.

1 $$T_{mrt} = \left[ {(T_{g} + 273)^{4} + \frac{{1.10 \times 10^{8} N_{a}^{0.6} }}{{\varepsilon D^{0.4} }}(T_{g} - T_{a} )} \right]^{1/4} - 273$$

After calculating the thermal comfort indices, three approaches were applied to determine the thermal benchmarks: linear regression, quadratic regression, and Probit analysis.

### EEG data processing and statistical analysis

The analysis of neural and physiological data followed a rigorous computational pipeline to ensure high signal-to-noise ratios, particularly given the challenges of outdoor data collection. Raw EEG signals were processed in MATLAB using the EEGLAB toolbox through a sequence that began with band-pass filtering from 0.5 to 45 Hz and notch filtering at 50 Hz to eliminate drift and powerline interference. To address channel-level artifacts, bad channels were identified based on a probability threshold of ± 3 standard deviations from the mean and subsequently interpolated. Following re-referencing to the common average (CAR), strict artifact rejection was implemented before Independent Component Analysis (ICA) decomposition by automatically removing segments with muscle activity or gross movements exceeding 100 µV. Ocular and electromyographic artifacts were further isolated and manually rejected via ICA components that correlated with blinks or high-frequency spectra. Following this rigorous cleaning procedure, an average of 12.8% (SD = 4.2%) of EEG data segments across all participants and conditions were rejected due to residual artifacts, a rate comparable to those reported in similar mobile EEG studies^13,22^. This rejection rate was balanced across experimental conditions, minimizing potential bias. This cleaned dataset was then segmented into stimulus-locked epochs and baseline-corrected to facilitate the calculation of power spectral density across the delta, theta, alpha, beta, and gamma bands. Parallel to the neural analysis, physiological metrics were refined by deriving heart rate variability indices from interbeat intervals and calculating weighted average skin temperatures through the application of Eq. (2).

2 $$T_{{{\mathrm{overall}}}} = 0.15T_{{{\mathrm{forehead}}}} + 0.19T_{{{\mathrm{chest}}}} + 0.10T_{{{\mathrm{upper}}\;{\mathrm{arm}}}} + 0.19T_{{{\mathrm{lower}}\;{\mathrm{back}}}} + 0.37T_{{{\mathrm{upper}}\;{\mathrm{leg}}}}$$

The normality of the distribution for all continuous dependent variables (EEG band powers, HR, SKT, TSV, etc.) was assessed using the Shapiro–Wilk test. As most variables met the assumption of normality (*p* > 0.05), parametric tests were employed. Statistical analyses were performed using SPSS (version 26.0). Descriptive statistics (mean ± SD) were calculated for all variables. A repeated-measures ANOVA was employed to assess the effects of experimental conditions and SVF levels on neurophysiological and subjective responses, with Greenhouse–Geisser corrections applied where sphericity was violated. Post-hoc comparisons were conducted using the Bonferroni test to identify specific differences between groups. Furthermore, the relationship between neurophysiological features and thermal sensation was evaluated using Pearson correlation coefficients. For all analyses, the statistical significance threshold was set at *p* < 0.05. To control for the risk of Type I errors due to multiple comparisons across the five EEG frequency bands, five physiological variables, and five SVF conditions, the False Discovery Rate (FDR) procedure was applied to the *p*-values of the primary repeated-measures ANOVA and post-hoc comparisons. Only results that remained significant after FDR correction (q < 0.05) are reported as statistically significant.

## Results

### Neural and physiological responses to visual stress and subsequent green recovery

**Fig. 3:**
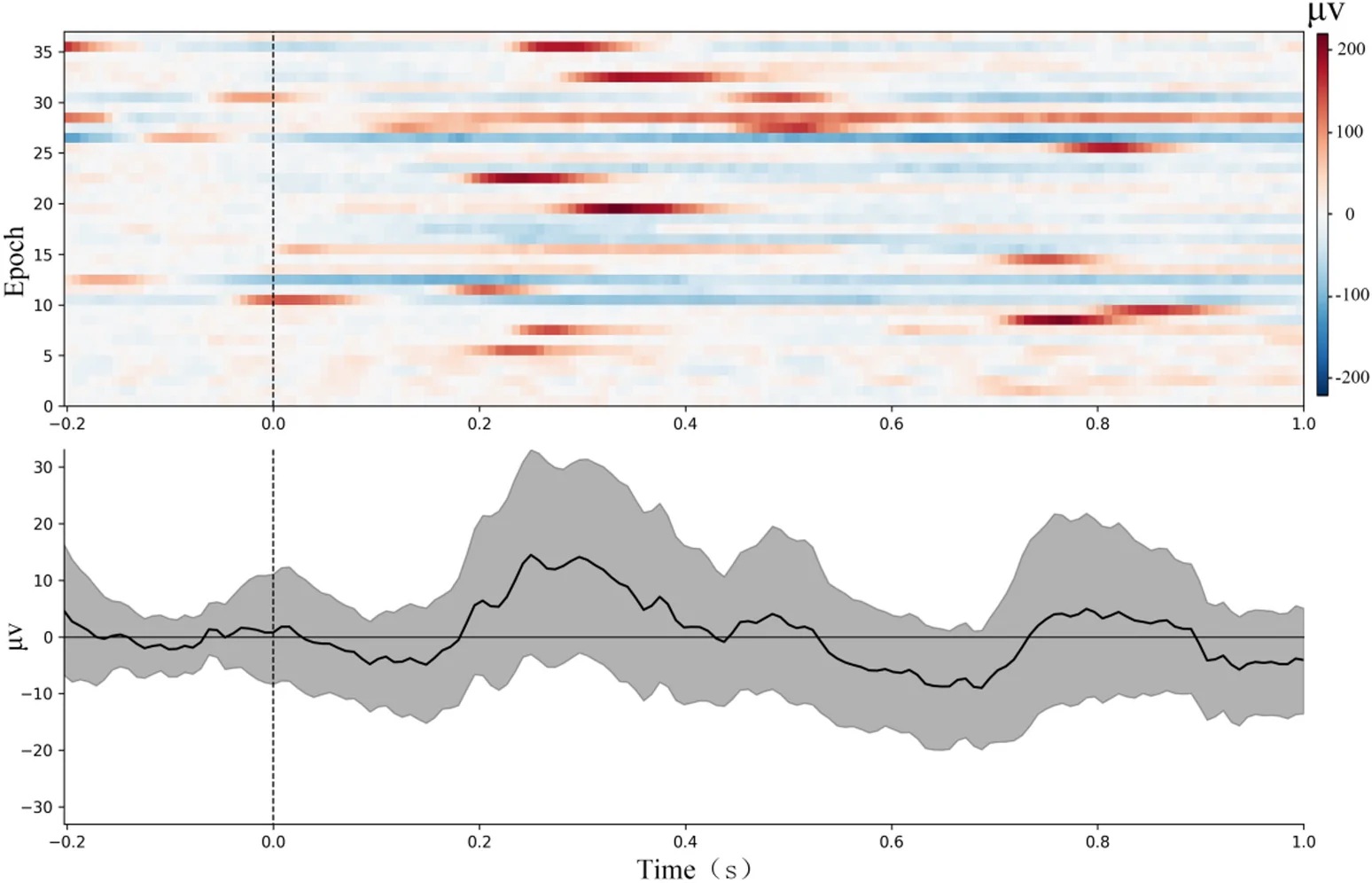
Heatmap of spatiotemporal EEG responses at AF3 channel during stress task phase.

**Fig. 4:**
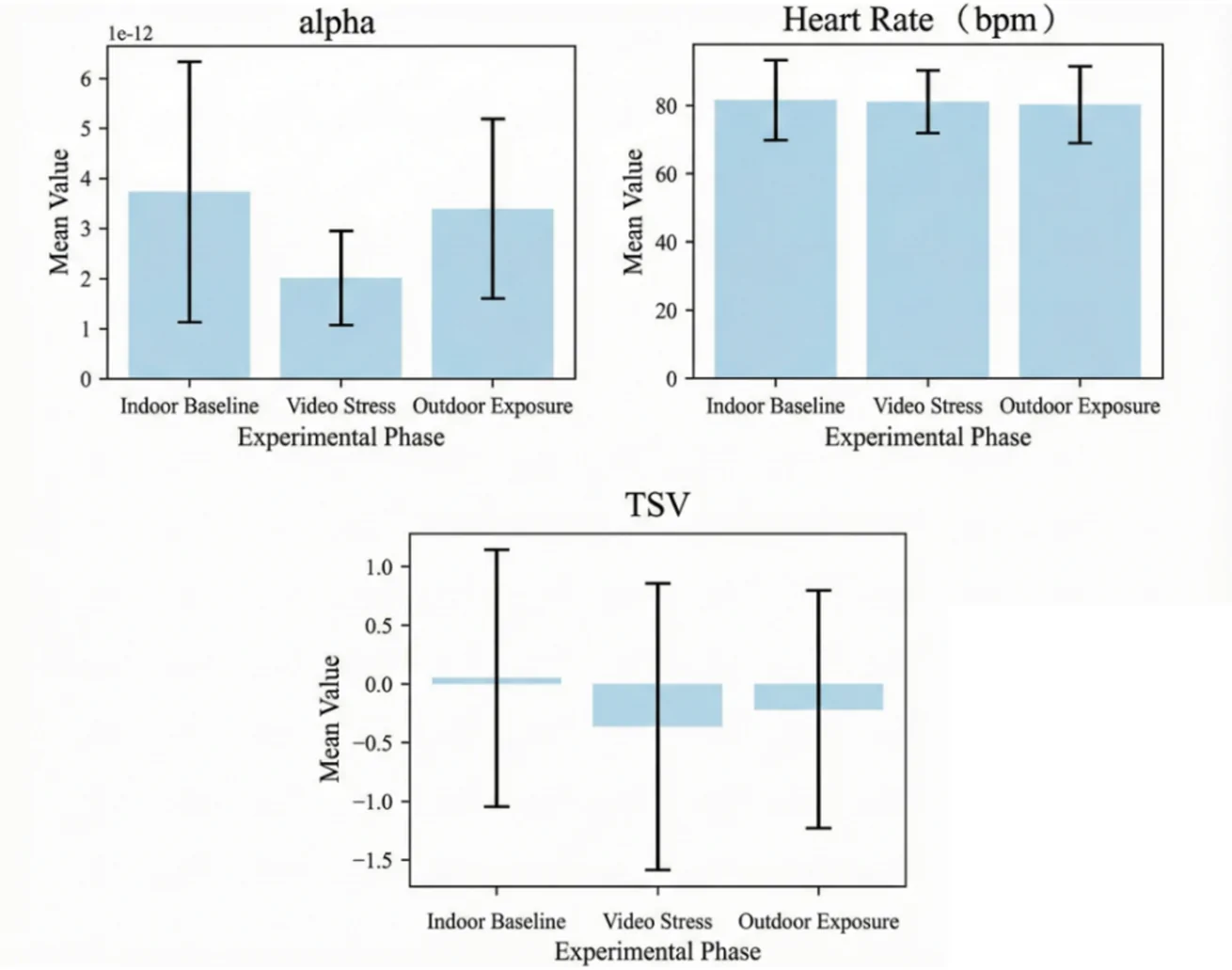
Comparison of alpha wave, HR and thermal sensation values across experimental phases.

Exposure to the emotionally arousing red video successfully induced acute psychophysiological arousal, eliciting significant and rapid changes in central and autonomic nervous system activity. As illustrated in Fig. 3, time–frequency analysis of EEG signals at channel AF3 was selected as a representative prefrontal channel due to its established role in emotional and cognitive arousal monitoring^5^. This channel revealed a marked suppression of alpha power (8–13 Hz) during the stress phase, accompanied by increased beta (13–30 Hz) and gamma (30–45 Hz) oscillatory activities, reflecting heightened cognitive arousal and sympathetic activation.To ensure the representativeness of these findings, Fig. 4 illustrates the grand average waveform derived from the entire 15-min exposure period. The continuous data were segmented into 1-s epochs, which were then averaged across all participants for each SVF scenario to provide a standardized comparison of the periodic physiological oscillations under different environmental stimuli. However, heart rate did not significantly change during the visual stimulation phase (Baseline HR: 81.4 ± 11.6 bpm; Stress HR: 81.0 ± 9.1 bpm; t(40) = 0.40, *p* = 0.694, Cohen’s d =  − 0.06), indicating that the audiovisual stressor may have induced cognitive engagement without robust autonomic arousal in this sample (Fig. 4).

**Fig. 5:**
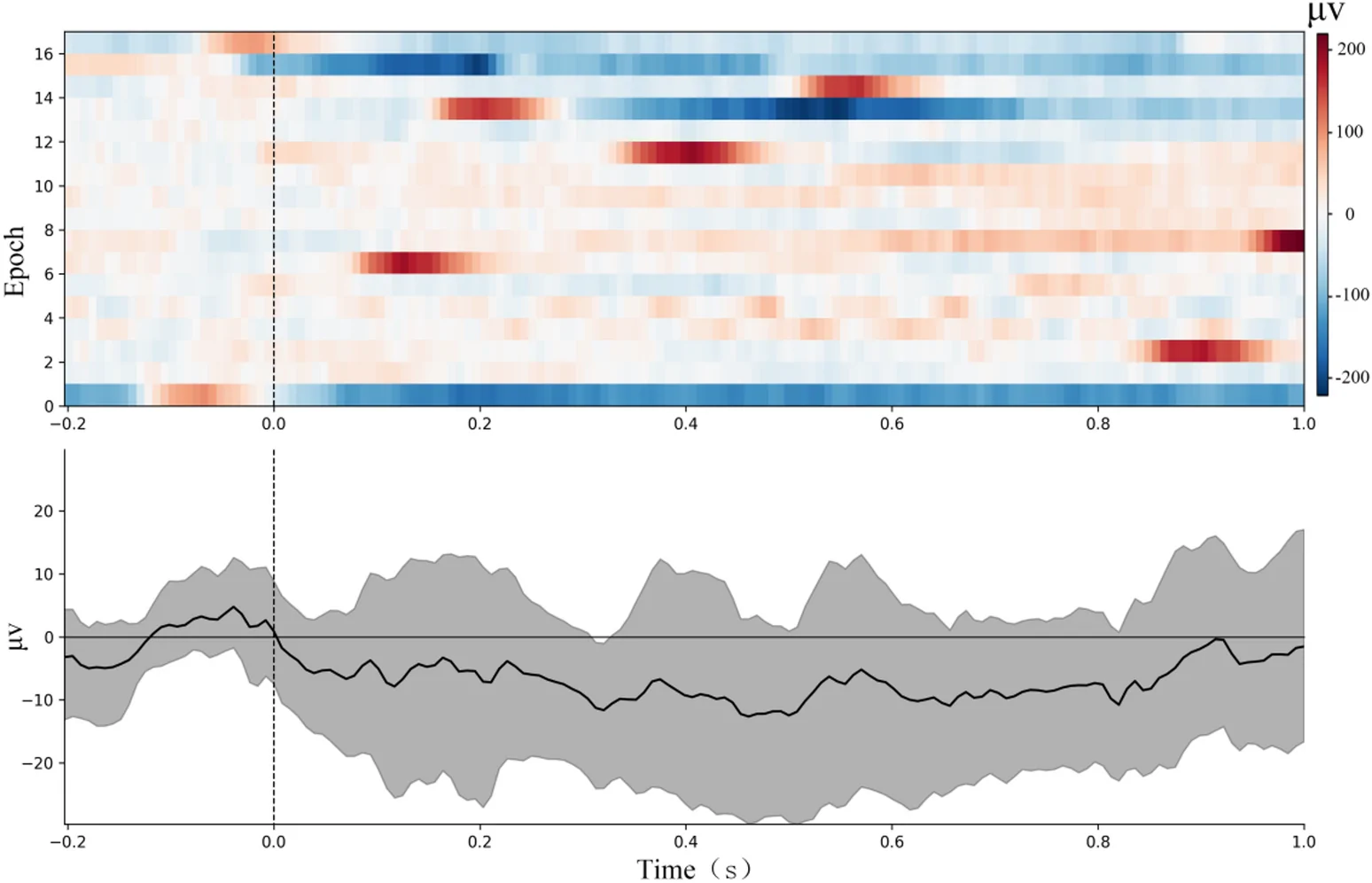
Heatmap of spatiotemporal EEG responses at AF3 channel during green landscape recovery phase.

**Fig. 6:**
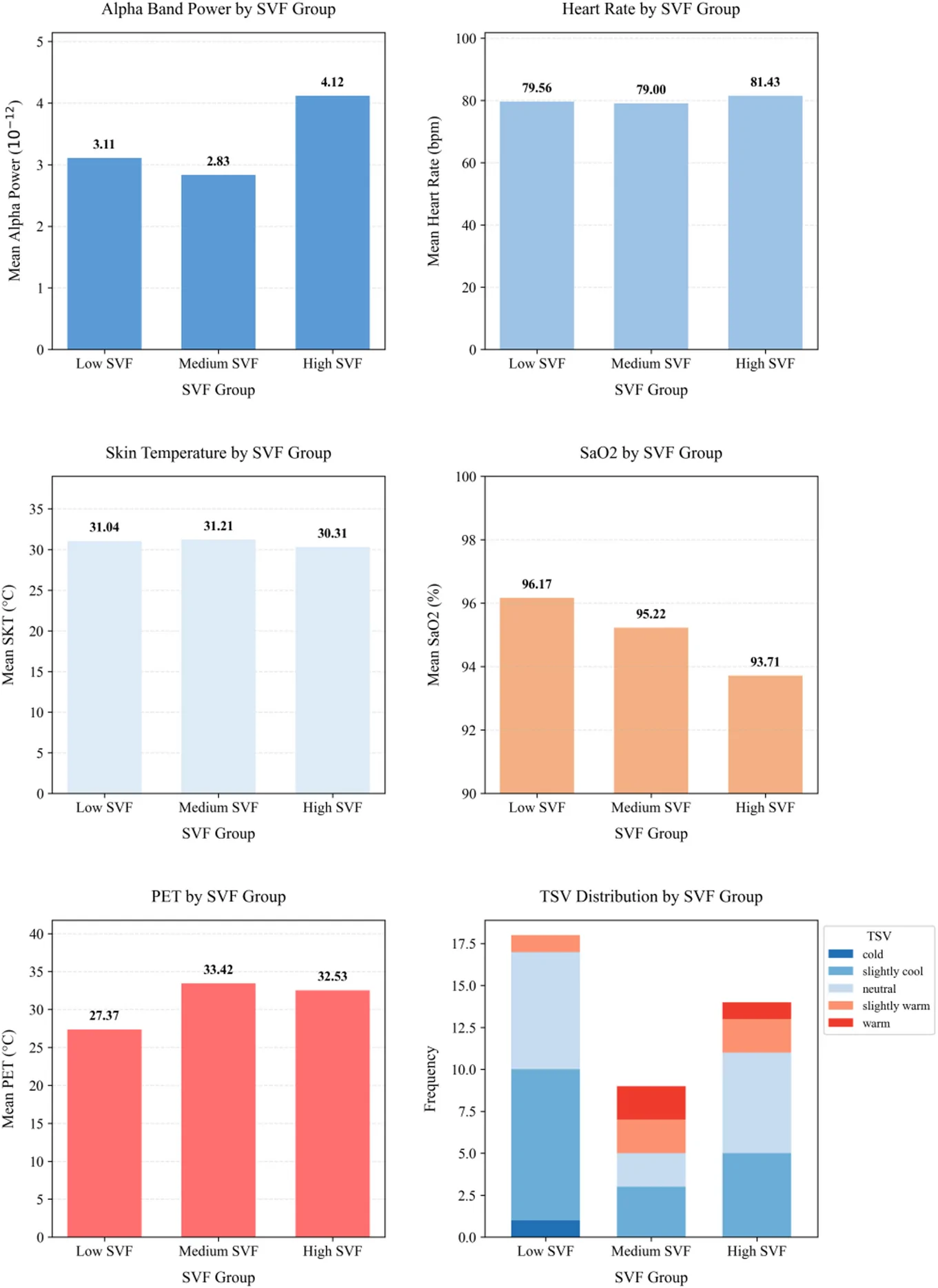
Comprehensive comparison of neurophysiological and environmental thermal parameters by SVF intervals.

Subsequent exposure to green landscapes, particularly those with low SVF (e.g., enclosed tree-canopy settings, SVF ≈ 0.073), facilitated pronounced neural and physiological recovery. Figure 6 demonstrates a substantial restoration of alpha power during the recovery phase, indicating a return to a relaxed yet alert state. Concurrently, heart rate showed a decreasing trend (Fig. 5), suggesting a potential reinstatement of autonomic balance, though statistical significance was not assessed for this phase. Subjective reports aligned closely with these physiological trends: participants reported reduced stress and improved thermal comfort following green recovery, as quantified by PANAS and TCV scores.

Topographic maps of EEG activity further illustrated the spatial dynamics of this response, with prefrontal and parietal regions showing the most pronounced changes in alpha and beta power across conditions. Importantly, the recovery effect was most evident in environments with higher visual enclosure and natural elements, underscoring the role of visual input in modulating neurophysiological state.

Together, these findings confirm that the visually arousing stimulus triggers rapid neuroautonomic arousal, while exposure to restorative green environments promotes recovery, as captured through synchronous EEG, physiological, and subjective measures.

### Differential neuro-physiological responses across SVF gradients​

Analysis of neurophysiological signals revealed partially distinct responses to variations in SVF, with low-frequency oscillations (Delta/Theta) and high-frequency bands (Beta/Gamma) showing systematic changes, while Alpha power remained stable across SVF levels.

**Fig. 7:**
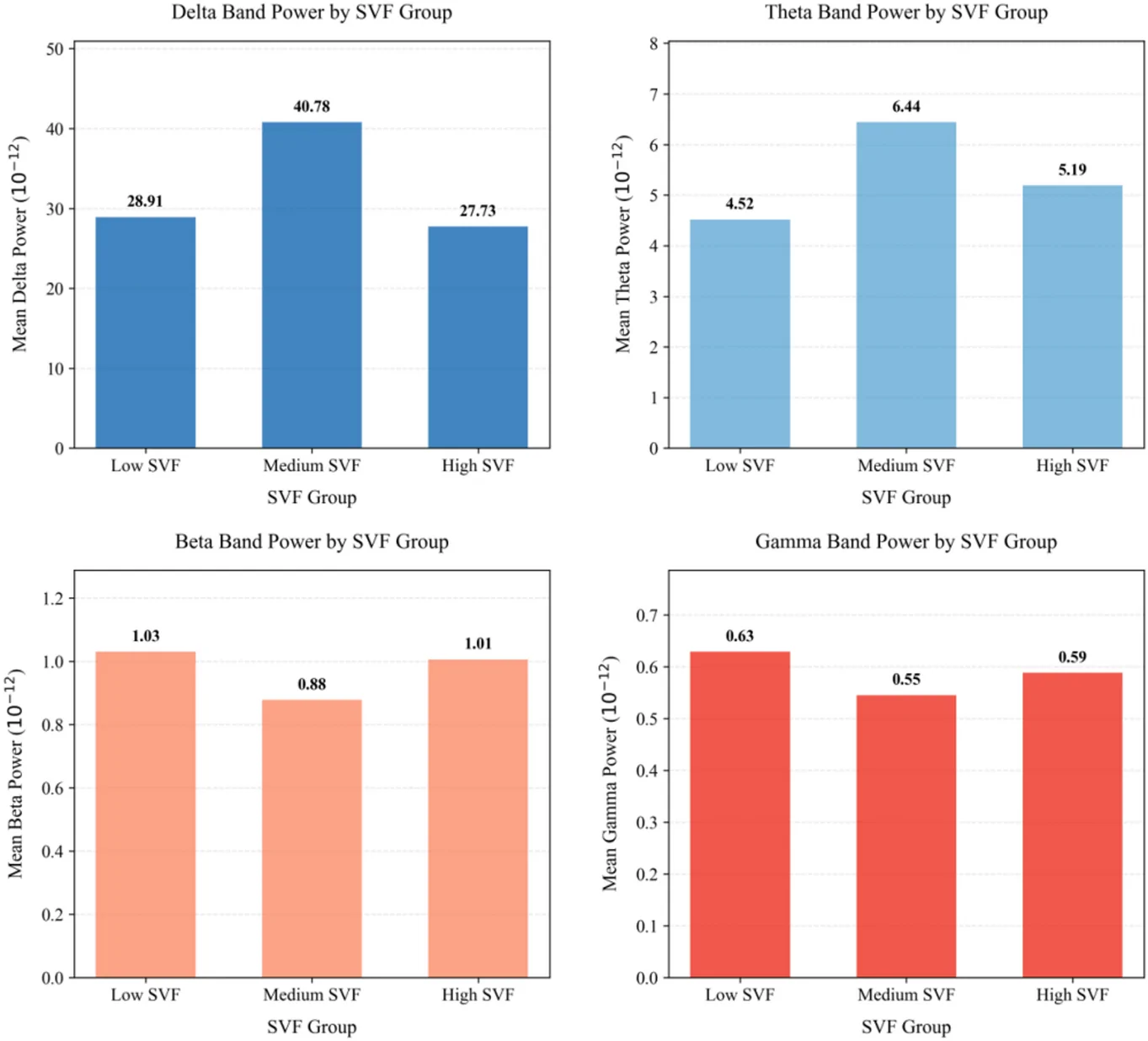
Comparison of EEG band power across different sky view factor (SVF) groups.

As demonstrated in Figs. 6 and 7, neural oscillatory power exhibited distinct patterns across different SVF environments. Contrary to the expectation that open spaces simply increase high-frequency activity, Medium SVF environments elicited the highest low-frequency power, characterized by peak Delta (40.78 × 10^−12^) and Theta (6.44 × 10^−12^) activities, suggesting a state of deep internalization or drowsiness. A one-way ANOVA revealed no significant main effect of SVF on alpha power (F(2, 38) = 1.89, *p* = 0.165, η2 = 0.090). However, a post-hoc comparison using Welch’s t-test showed a marginal trend for higher alpha power in High SVF (4.12 × 10^−12^ ± 2.14 × 10^−12^) compared to Medium SVF (2.83 × 10^−12^ ± 9.61 × 10^−13^; t(21) = 1.96, *p* = 0.065), suggesting a potential effect that may become significant with increased statistical power. Meanwhile, high-frequency oscillations (Beta and Gamma) reached their lowest points in Medium SVF conditions (0.88 × 10^−12^ and 0.55 × 10^−12^, respectively), recovering slightly in High SVF settings (1.01 × 10^−12^ and 0.59 × 10^−12^). These differential EEG patterns were corroborated by heart rate measures, where High SVF showed the highest mean Heart Rate (81.43 bpm) compared to Low SVF (79.56 bpm), though no statistical test was performed for this comparison.

Our analysis demonstrated that SVF differentially altered the microclimate and subsequently shaped occupants’ thermal perception. PET increased from 27.37 °C under Low SVF to a peak of 33.42 °C under Medium SVF, before slightly decreasing to 32.53 °C under High SVF conditions. Mean Skin Temperature (SKT) remained relatively stable at approximately 31 °C under Low and Medium SVF conditions but dropped to 30.31 °C under the highest SVF. SpO₂ showed a gradual decline with increasing openness, decreasing from 96.17% in Low SVF to 93.71% in High SVF. Regarding thermal perception, the dominant sensation shifted from “neutral” and “cool” under Low SVF to a combination of “slightly warm” and “warm” under Medium and High SVF (Fig. 7).

Subjectively, the physiological data aligns with the reported thermal sensations, where increased PET in Medium and High SVF corresponded with warmer thermal votes. The observed decrease in SKT at the highest SVF may be attributed to increased convective heat loss or sweat evaporation in open areas. These results demonstrate that SVF modulates neurophysiological states in a pattern-specific manner; notably, Medium SVF environments appear to facilitate low-frequency neural dominance (Delta/Theta), whereas High SVF promotes alpha-related relaxation coupled with elevated cardiovascular activity.

### Coupling between subjective reports and neurophysiological indicators

**Fig. 8:**
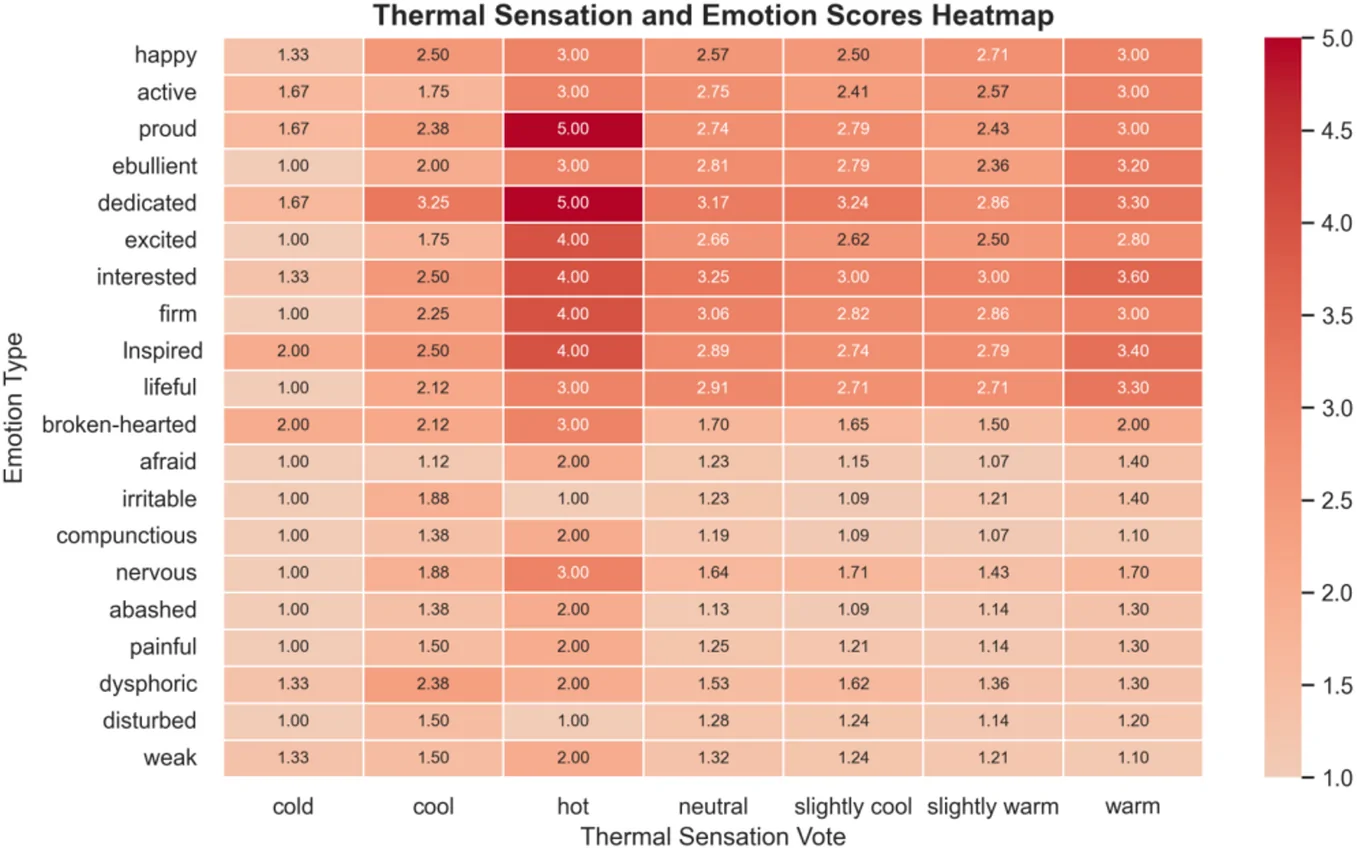
Heatmap of association between thermal sensation votes and emotion scores.

As quantified through correlation heatmaps (Fig. 8), positive affective states such as “happy” and “relaxed” showed no significant correlation with alpha power or heart rate, whereas negative emotions including “irritated” and “uncomfortable” correlated with elevated beta and gamma power and increased heart rate.

Subjective evaluations revealed a highly significant positive correlation between TSV and TCV (r = 0.85, *p* < 0.01), confirming that the “neutral” to “slightly warm” range was perceived as the thermal comfort zone in the studied urban landscapes. Positive affect showed no significant correlation with alpha power (r =  − 0.07, *p* = 0.675, FDR q = 0.675) or with heart rate (r =  − 0.11, *p* = 0.485, FDR q = 0.675), suggesting that the relationship between emotional state and neurophysiological markers may be more complex than initially hypothesized.

Notably, the relationship between TSV and EEG biomarkers revealed that showed a tendency toward decreased alpha power and increased high-frequency oscillations, particularly over frontal and temporal regions. These results demonstrate that the multimodal framework can effectively capture the “Brain-Body-Environment” interaction, where neural oscillations serve as a sensitive internal proxy for conscious comfort perception.

**Fig. 9:**
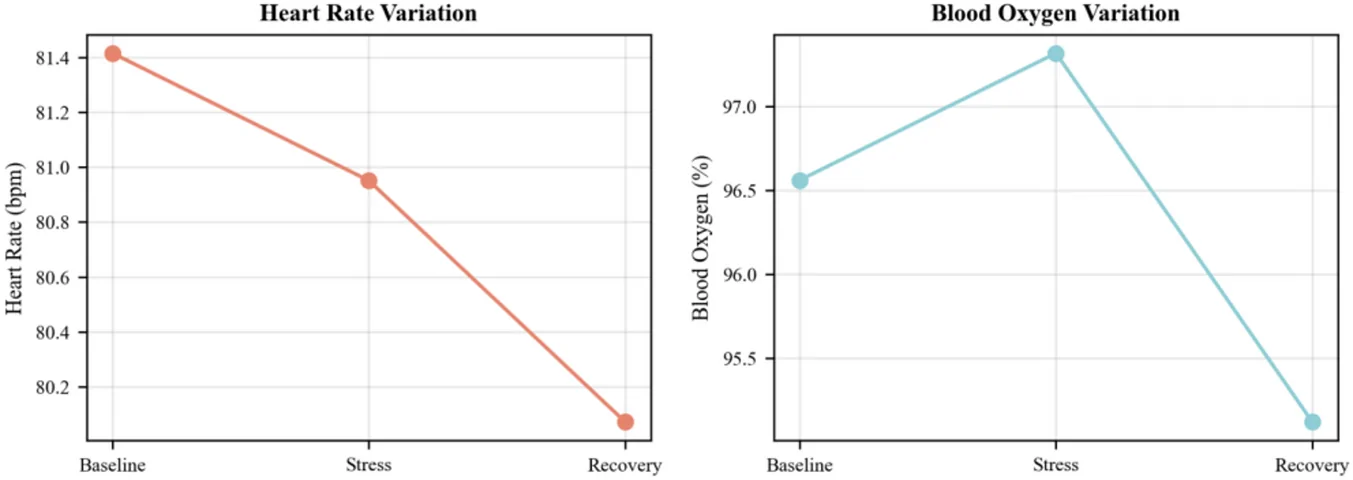
Dynamic patterns of heart rate and blood oxygen during baseline, stress and recovery phase.

The distribution of emotional responses across different thermal sensation categories, showing that positive emotions were most frequently reported in neutral conditions, while negative emotions clustered around extreme cold or hot sensations. These subjective reports align closely with the patterns of physiological arousal shown in heart rate and skin temperature measurements (Fig. 9).

**Fig. 10:**
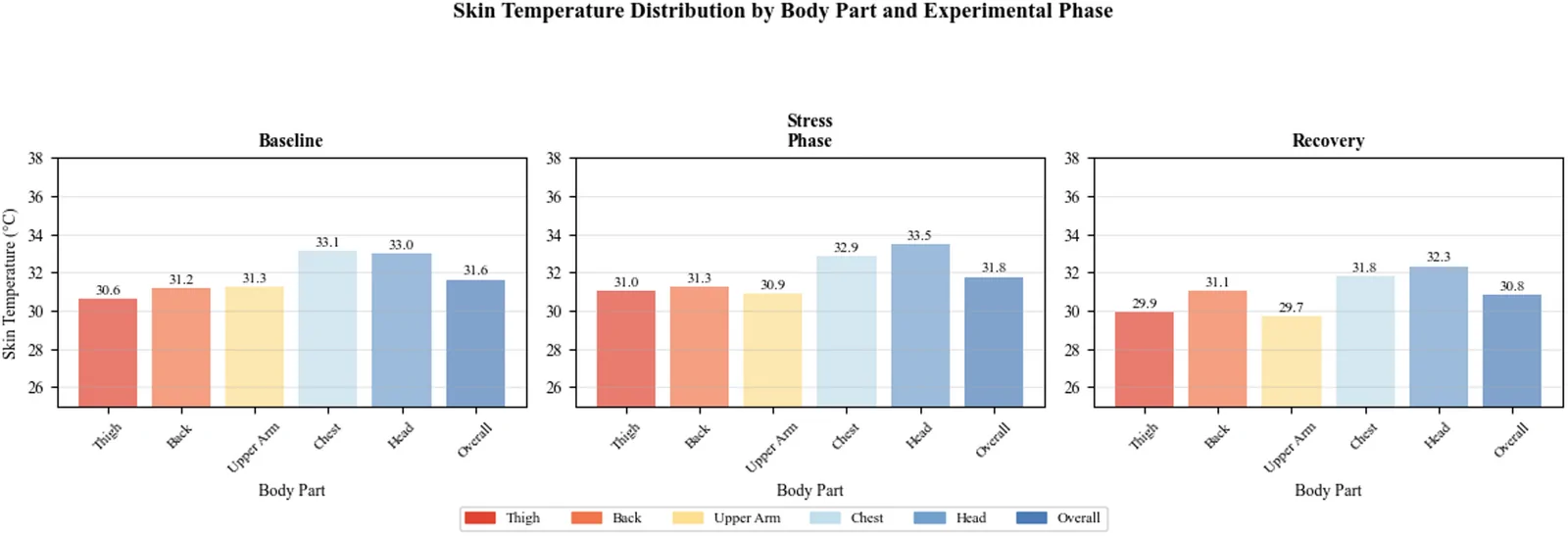
Skin temperature distribution by body part and experimental phase.

Further analysis of the overall distribution of skin temperature in different body parts shows that during the baseline phase, the temperatures of the upper leg, back, upper arm, chest, head, and overall body were 30.6 °C, 31.2 °C, 31.3 °C, 33.1 °C, 33.0 °C, and 31.6 °C respectively; during the pressure phase, they were 31.0 °C, 31.3 °C, 30.9 °C, 32.9 °C, 33.5 °C, and 31.8 °C respectively; and during the recovery phase, they were 29.9 °C, 31.1 °C, 29.7 °C, 31.8 °C, 32.3 °C, and 30.8 °C respectively. The skin temperature bar chart also intuitively displays the temperature differences of each body part in different phases. For example, the forehead temperature was 33.0 °C during the baseline phase and 32.9 °C during the pressure phase, dropping to 32.3 °C during the recovery phase; the chest temperature was 33.1 °C during the baseline phase and 33.5 °C during the pressure phase, and 31.8 °C during the recovery phase (Fig. 10).

## Discussion

### The dual pathways of SVF: from visual input to central neural modulation

Our findings reveal that SVF influences human thermal perception through two intertwined pathways: a well-established microclimatic pathway and a composite SVF-modulated pathway that integrates both visual and associated thermal inputs. It is essential to acknowledge the inherent collinearity between SVF, solar radiation, and Tmrt in field studies. Consequently, the identified “neural response” is best interpreted as the brain’s integrated processing of the total environmental cue—the “visual-thermal gestalt”—of an open or enclosed space. Beyond its established role in modulating solar radiation and convective heat exchange (as quantified by PET and UTCI), SVF exerts a direct effect on central nervous system activity via visual input. Medium SVF environments, characterized by the highest PET, were associated with elevated Delta and Theta oscillatory activity, indicating a transition toward drowsiness or deep internalization likely triggered by thermal load. In High-SVF environments (> 0.8), the neurophysiological landscape was characterized by relatively higher Alpha power compared to Beta/Gamma oscillations, although statistical analysis revealed no significant main effect of SVF on alpha power across conditions. While Beta and Gamma oscillations exhibited a slight recovery compared to their nadir in Medium-SVF conditions, their absolute power remained subordinate to the dominant Alpha activity Fig. 7. This suggests that the visual openness of large plazas facilitates a state of 'relaxed wakefulness,' where the brain maintains awareness without the heightened cortical arousal or cognitive stress typically indicated by elevated Beta/Gamma levels. Notably, this neural relaxation persists even when physiological arousal (heart rate) remains high, highlighting a distinct decoupling between visual relaxation and autonomic response. This finding challenges the conventional view that open spaces solely trigger alertness, pointing instead to a complex psychophysiological state regulated by visual openness.

This neural modulation occurred within the first few minutes of exposure, demonstrating the rapidity of visual processing in environmental appraisal. The observed anteriorization of alpha activity in low-SVF conditions particularly implicates prefrontal regulatory mechanisms, potentially through top-down attention modulation and emotional appraisal processes. Furthermore, the maintained functional connectivity between occipital and frontal regions during green exposure suggests a neural mechanism for the restorative effects of natural environments, facilitating the transition from effortful attention to relaxed reflection.

These results align with growing evidence in environmental neuroscience that spatial visual characteristics evoke predictable neurophysiological responses, extending understanding of urban design’s impact beyond thermodynamic factors. The microclimatic role of SVF is corroborated by studies in Harbin, where SVF strongly correlates with thermal comfort indices such as PET, and vegetation shading in street canyons reaches 56.3% efficiency^23^. Diurnal studies in Beijing further show that SVF effects vary temporally: daytime cooling is dominated by building and vegetation shade, whereas nocturnal warming is influenced by anthropogenic heat and albedo^24^. This supports our observation that a high SVF can simultaneously exacerbate solar radiation and promote convective cooling. Importantly, SVF’s thermal influence is nonlinear. While shading effects saturate at very low SVF values, heat retention continues to rise, underscoring the need for context-aware design^23–25^.

From the neural perspective, studies in Chengdu indicate that Green View Factor and Sky VF alleviate emotional arousal and foster positive affective states, consistent with our findings of alpha enhancement under low SVF^6^. Such effects resonate with Attention Restoration Theory and Stress Reduction Theory, suggesting that natural elements engage prefrontal regulation, as reflected in alpha anteriorization^6^. The enhanced occipitofrontal functional connectivity may underpin the alerting effects of high-SVF settings via integrated visual-emotional processing^6,26^.

Critically, these pathways are synergistic. For example, building height can yield daytime cooling (microclimatic effects) but nocturnal heat retention, while concurrently evoking visual oppression that may suppress alpha activity (neural effects)^24^. Therefore, urban design must integrate both physical and psychological dimensions of SVF to optimize human thermal and perceptual comfort.

### Coordinated neuro-physiological responses to environmental stimuli

Medium SVF environments, characterized by a significant thermal load (peak PET), were associated with elevated Delta and Theta oscillatory activity. While such increases are often linked to drowsiness or internally focused states, an alternative explanation is that this pattern reflects a state of reduced cortical arousal or fatigue in response to prolonged thermal strain, rather than a specific “protective shutdown” mechanism. Similarly, the numerically higher Alpha power observed in High SVF environments (though not statistically significant) might be consistent with relaxed wakefulness, but we cannot rule out contributions from altered visual attention or eye closure patterns, which are known to modulate alpha rhythms. Thus, High SVF spaces induce a state of physiological arousal with relatively higher alpha oscillations (though not statistically significant), distinct from the “Alertness” hypothesis traditionally associated with open environments. Crucially, this heightened cortical arousal co-occurs with thermal discomfort (High TSV). This suggests that urban design must address both the “Thermal Stress” (via shading) and the “Visual Stress” (via greenery) simultaneously to prevent a compounded negative effect on the user. While our study demonstrates the convergent validity of these multimodal signals (e.g., environments eliciting high alpha power showed a trend toward lower heart rate, but no significant correlation with positive affect was found), the integration remains at a descriptive, correlational level. We acknowledge that we did not implement formal data fusion techniques (e.g., canonical correlation analysis or machine learning-based predictive models) to quantify how these signals jointly explain thermal perception. The primary contribution of this study is to establish the foundational cross-system coupling (the “EEG-Skin-Heart” axis) in response to SVF gradients. Future research should build on these findings by applying advanced multivariate models to predict individual comfort states from the combined neurophysiological signature.

### Implications for theory and healthy city design

Our study theoretically advances thermal comfort research beyond conventional steady-state models like Fanger’s PMV, which, as we note in the introduction, has limited applicability in dynamic outdoor settings. By incorporating neural markers, our findings provide a mechanistic complement to adaptive thermal comfort models and indices such as UTCI, suggesting that subjective comfort arises from an interaction between peripheral thermal load (captured by UTCI) and central nervous system appraisal (captured by EEG). This framework aligns with and extends the growing recognition that urban three-dimensional morphology, particularly SVF, influences thermal environments through complex pathways that integrate physical, physiological, and psychological dimensions^27^. The demonstrated role of central nervous processes in thermal appraisal suggests that comfort models should account for individual differences in neural processing styles and stress vulnerability. Furthermore, the time-sensitive nature of neurophysiological responses supports the development of dynamic comfort models that capture real-time adaptation processes, moving beyond steady-state predictions. This approach is corroborated by research indicating that physiological parameters like heart rate variability and skin temperature are sensitive to autonomic nervous system activity and can serve as objective indicators of thermal comfort states, providing a physiological basis for real-time monitoring^28^.

Practically, these insights advocate for SVF-aware landscape design, which optimizes shading and visual enclosure in high-heat areas to promote both thermal and neural comfort. Urban designers can leverage these findings to create restorative spaces that mitigate thermal strain not only microclimatically but also neurophysiologically. For example, this can be achieved through strategically vegetated corridors or visually sheltered rest areas, approaches supported by studies in Beijing which found that dispersed green spaces with complex morphologies yield better cooling effects (1.4–2.2 °C reduction) than centralized ones^29^. High-resolution thermal observations from UAV studies in Wuhan further confirm that SVF is a crucial warming factor at fine scales for impervious surfaces, with an efficiency threshold around 0.7, and that shade casting by 3D elements is a core mechanism for outdoor heat dissipation^7^. The significant association of heart rate identified in our study suggests that real-time physiological monitoring could serve as a valuable input for adaptive environmental control systems, enabling buildings and urban spaces to respond dynamically to occupants’ neurophysiological states. This aligns with research in Guangzhou’s parks using deep learning and panoramic imagery, which found that environments with higher naturalness (often associated with lower SVF and higher green view index) promote positive perceptions like attraction and naturalness, while reducing negative feelings like suppression^30^. Moreover, studies of medium-sized city parks highlight that combining low-albedo pavements with broad-canopy, high-trunk trees can achieve optimal thermal comfort, emphasizing the synergy between material properties and vegetation structure that our neuro-physiological framework encapsulates^31^.

Ultimately, integrating SVF’s dual pathways into design allows for creating alertness-promoting spaces with higher SVF where needed, and restorative, relaxing spaces with lower SVF and strategic vegetation, enabling multi-dimensional optimization of human comfort^27,29^.

### Practical implications and design guidelines

**Fig. 11:**
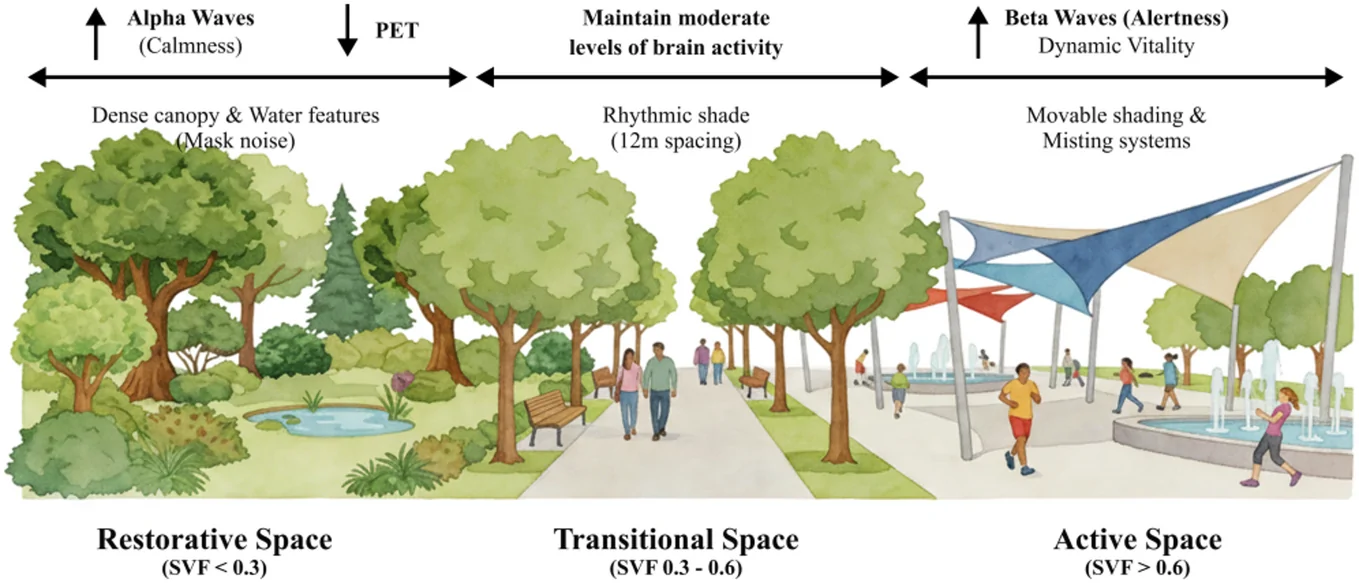
SVF-based landscape design guidelines.

The results of this study not only reveal the mechanism by which SVF affects thermal comfort through neurophysiological pathways, but also provide directly actionable basis for sustainable urban landscape design. Based on the above findings, we propose the following design matrix (as shown in Table 3 and Fig. 11):

**Table 3:** SVF-based design matrix for brain-informed landscape interventions targeting multi-dimensional comfort.

City space types	Thermal environment & neuro-comfort goals	Recommended SVF range	Key landscape design strategies
Restorative Spaces (e.g., Park Quiet Areas, Therapeutic Gardens)	Maximize psychological relaxation and physiological cooling.​​ Goal: Lower sympathetic nerve activity (reflected by HRV), enhance EEG alpha/theta waves associated with calmness, and significantly reduce PET/UTCI	Low (e.g., < 0.3)	1. Vegetation Strategy: Plant tall, dense-canopied trees (e.g., ginkgo, locust) to form a highly enclosed forest-understory space. Combine with multi-layered shrubs and ground cover to increase Green View Index (GVI) and ecological richness^32^ 2. Sensory Design: Incorporate gentle, continuous sound water features (e.g., streams, small waterfalls) to mask urban noise and provide auditory restoration. Use natural materials like wood and stone for pathways and benches to enhance tactile connection 3. Spatial Layout: Create intimate, secluded “garden rooms” using vegetation, terrain, or low fences to enhance the sense of being away and security. Set up pavilions or pergolas to provide all-weather, stable shade^33^
Transitional Spaces​​ (e.g., Tree-lined Avenues, Plaza Edges)	Balance visual alertness and thermal comfort Goal: Maintain moderate levels of brain activity (avoiding excessive beta waves), ensure smooth physiological transition, and achieve a neutral thermal sensation (TSV)	Moderate (e.g., 0.3–0.6)	1. Spatial Patterning: Adopt sparse forest lawns and tree array plazas to create a rhythm of dappled light and shade. This meets the need for both openness and shade protection ^34^ 2. Vegetation Configuration: Optimize the spacing of street trees. A spacing of around ​12 m​has been shown to effectively balance shade provision and ventilation needs, significantly reducing Mean Radiant Temperature (MRT)^25^ 3. Functional Integration: Design rest spots at regular intervals with comfortable, back-supported seating, allowing people to pause and briefly recover. This is particularly important for elderly and heat-sensitive individuals
Active Spaces (e.g., Main Plazas, Sports Fields)	Maintain visual openness and vitality while mitigating heat stress Goal: Allow for higher arousal levels (accepting transient increases in beta waves) but prevent severe thermal discomfort (TSV > + 1.5) and excessive physiological strain	High​​ (e.g., > 0.6)	1. Dynamic Cooling: Install dynamic water features such as fountains, misting systems, or interactive splash pads. Their evaporative cooling effect can significantly lower the local PET, while their dynamic visual and auditory stimuli enhance the environment’s vitality^33^ 2. Flexible Shading: Use movable shading facilities such as large shade sails, retractable awnings, or open pergolas. This provides adjustable shade that can be deployed during peak radiation hours (noon/afternoon) and retracted at other times to allow for nighttime heat release^33^ 3. Permeable Ground: Use high-albedo, permeable paving materials (e.g., light-colored permeable bricks, grass pavers) to reduce heat storage and surface temperatures, while also aiding in rainwater management^25,33^

### Limitations and future perspectives

The generalizability of the current findings is primarily constrained by the homogeneity of the participant pool, which consisted exclusively of healthy young adults. This limits the external validity of our conclusions, as thermal perception and physiological regulation—particularly autonomic and central nervous system responses—are known to vary substantially across age groups (e.g., older adults), health conditions (e.g., individuals with cardiovascular or thermoregulatory disorders), and levels of acclimatization. Future research should therefore encompass a broader demographic spectrum, especially heat-vulnerable populations, to validate these neurophysiological patterns.

Regarding environmental characterization, the current study strategically focused on contrasting SVF extremes to capture pronounced physiological responses. However, this approach resulted in a lack of intermediate SVF levels, specifically within the 0.4–0.6 range. Expanding the SVF gradient in future work would provide a more granular understanding of the non-linear relationship between sky exposure and thermal comfort. Furthermore, while the 14-channel EEG system offered a practical balance between portability and data quality, employing high-density arrays could enhance source localization and the suppression of myogenic artifacts.

A critical challenge remains in the full decoupling of visual and thermal stimuli. Although VR was utilized to standardize visual input, the inherent covariance between SVF, solar radiation, and mean radiant temperature suggests that the observed “SVF → EEG → HR → TSV” pathway likely reflects a composite environmental effect. Future experiments conducted within climate chambers coupled with VR would allow for the isolated manipulation of these variables. Moreover, while factors such as vegetation type and wind speed were noted, their potential confounding influence, alongside variations in clothing insulation and individual acclimatization, merits more rigorous control in longitudinal or cross-cultural investigations. Integrating neural markers with eye-tracking and machine learning-based personalized models represents a promising trajectory. The development of robust, real-time EEG processing pipelines, as recently reviewed^35^, will be critical for translating these findings into adaptive environmental control systems and 'brain-informed’ urban interfaces. An additional limitation concerns the experimental design, where a 5-min interval occurred between the stressor offset and the start of the outdoor exposure. While participants remained seated in the indoor environment during this interval, which was necessary for logistical preparation, we cannot completely rule out that some spontaneous stress recovery may have occurred during this period. However, any such spontaneous recovery would be expected to be similar across all five subsequent SVF exposures. Therefore, the differential neurophysiological recovery patterns observed across the Low, Medium, and High SVF conditions can still be robustly attributed to the specific visual-thermal properties of the outdoor spaces, as they varied systematically while the prior indoor interval was held constant.

## Conclusions

This study employed a multimodal neurophysiological approach to investigate the mechanisms by which visual and thermal cues in landscape gardens shape human thermal perception. Our findings demonstrate that the SVF influences thermal comfort through both microclimatic pathways and the potential modulation of central nervous system activity, warranting further investigation. Medium SVF environments, characterized by limited shading and poor ventilation, imposed a significant thermal load, reflected in peak PET and elevated Delta/Theta activity, indicative of state of reduced cortical arousal or drowsiness or heat-induced drowsiness. In contrast, High SVF environments presented a unique neuro-physiological profile characterized by a distinct decoupling between central and autonomic responses. While the body responded with peak Heart Rate (indicating autonomic arousal) and decreased Skin Temperature (due to convective heat loss), the central nervous system did not exhibit the high Beta/Gamma activity typically associated with cognitive load. Instead, the relatively higher Alpha power (though not statistically significant across SVF levels) overturns the expectation that open spaces solely trigger stress responses, suggesting that visual openness may act as a psychological buffer. Thus, High SVF spaces induce a specific state of “Physiological Arousal with Neural Relaxation” (or “Relaxed Arousal”), distinct from the “Alertness” hypothesis traditionally associated with open environments. This confirms that outdoor thermal perception is not merely a passive physical reception but involves active central nervous processing. These findings advocate for a paradigm shift from conventional environmental-physiological models toward an integrated “environment-neuro-physio-psychology” framework. The tight coupling observed among neural dynamics, physiological responses, and subjective reports underscores the value of combining EEG, physiological monitoring, and subjective measures in environmental studies.

For urban design practice, this research provides evidence-based guidance for creating healthier built environments. By optimizing visual enclosure through vegetation and architectural design, cities can mitigate thermal discomfort microclimatically and neurophysiologically. Future research should explore individual differences in neural responses to environments and develop real-time comfort models that incorporate neurophysiological feedback for adaptive environmental control. Our findings offer scientific evidence for evidence-based urban planning. The SVF-informed design principles can be integrated into green infrastructure standards and building codes, particularly for creating climate-resilient and health-promoting neighborhoods. This contributes directly to the United Nations Sustainable Development Goals (SDGs), especially SDG 11 (Sustainable Cities and Communities) by enhancing urban environmental quality, and SDG 3 (Good Health and Well-being) by mitigating heat stress and promoting mental restoration.

## Data Availability

The data that support the findings of this study are available from the corresponding author upon reasonable request.
